# Fundus autofluorescence in the diagnosis and monitoring of acute retinal necrosis

**DOI:** 10.1186/s12348-015-0042-3

**Published:** 2015-06-23

**Authors:** Tyson SJ Ward, Ashvini K Reddy

**Affiliations:** Department of Ophthalmology, University of Virginia, 1300 Jefferson Park Avenue, Charlottesville, VA 22901 USA

**Keywords:** Acute retinal necrosis, Fundus autofluorescence

## Abstract

**Background:**

Acute retinal necrosis (ARN), a vision threatening viral retinitis, is often diagnosed and treated based on clinical findings. These clinical features have been well characterized by various imaging modalities, but not using fundus autofluorescence (FAF), a noninvasive method of evaluating the neurosensory retina and retinal pigment epithelium (RPE) based on the detection of endogenous fluorophores.

**Findings:**

A patient diagnosed with ARN was followed over a 10-month period to identify and document the fundus findings using FAF imaging. Pathological changes present at the level of the neurosensory retina and RPE in ARN can be detected and characterized using fundus autofluorescence imaging.

**Conclusions:**

The borders of disease activity in ARN correlate with high-contrast changes in autofluorescence patterns to facilitate monitoring of disease progression.

## Findings

### Background

Acute retinal necrosis (ARN) is a potentially blinding retinitis that was first reported in the 1970s [1,2]. It commonly occurs in immunocompetent individuals and is commonly associated with varicella zoster virus (VZV) and herpes simplex virus (HSV) [3]. The diagnosis of ARN is often made based on clinical features of anterior uveitis, vitritis, vasculitis, and peripheral areas of creamy white retinal necrosis. Secondary complications include rhegmatogenous retinal detachment, occlusive vasculopathy, neovascularization, vitreous hemorrhage, and phthisis bulbi [3].

The diagnostic clinical features of ARN previously described have been characterized by fundus photography, fluorescein angiography, optical coherence tomography [4], and electrophysiological studies [5].

In this report we describe variations in fundus autofluorescence (FAF) that were observed in a patient clinically diagnosed with ARN. FAF imaging is a noninvasive method of evaluating the neurosensory retina and retinal pigment epithelium (RPE) based on the fluorophores in these tissues [6]. Hyper-autofluorescence in FAF suggests an increase in fluorophores of the RPE [7] or reduced blocking of fluorophores by damaged outer retina [8]. Decreased autofluorescence in FAF suggests atrophy or loss of RPE fluorophores [7].

### Method

The patient was followed over 10 months with serial FAF images on both a Topcon 50EX retinal camera (Topcon, Paramus, NJ, USA) using an exciter filter of 585 nm and a barrier filter of 695 nm and a Spectralis HRA + OCT confocal scanning laser ophthalmoscope (Heidelberg Engineering, Heidelberg, Germany) using an excitation wavelength of 488 nm. Although there is a difference in excitation wavelengths between the two FAF modalities used, this does not affect imaging results as comparison in the brightness of FAF images are not recommended even when using the same imaging modality. Relative comparisons can only be made within the same image with regard to intensity of hyperautofluoresence. A previous study showed that the green-light FAF images (514 nm) are superior for the accurate analysis of small, central, pathologic changes, and for the determination of the central geographical atrophy lesion size. Using only blue-light FAF could lead to an over interpretation of the size of atrophic patches and the center involvement [9]. Our literature search does not reveal any publication regarding this difference in ARN patients. This research was approved by the IRB at the University of Virginia.

### Case description

A 64-year-old female with a history of mild leukopenia was referred with a 1 week history of progressively increasing floaters with ‘fogging’ of vision and photophobia in the left eye with no changes in the right eye. The patient reported a history of right-sided herpes zoster ophthalmicus 1 month prior for which she completed a 10 day course of famciclovir. Past medical history included chicken pox in preschool and genital herpes (diagnosed 22 years prior and treated with acyclovir). She denied a history of human immunodeficiency virus (HIV) disease.

**Figure 1:**
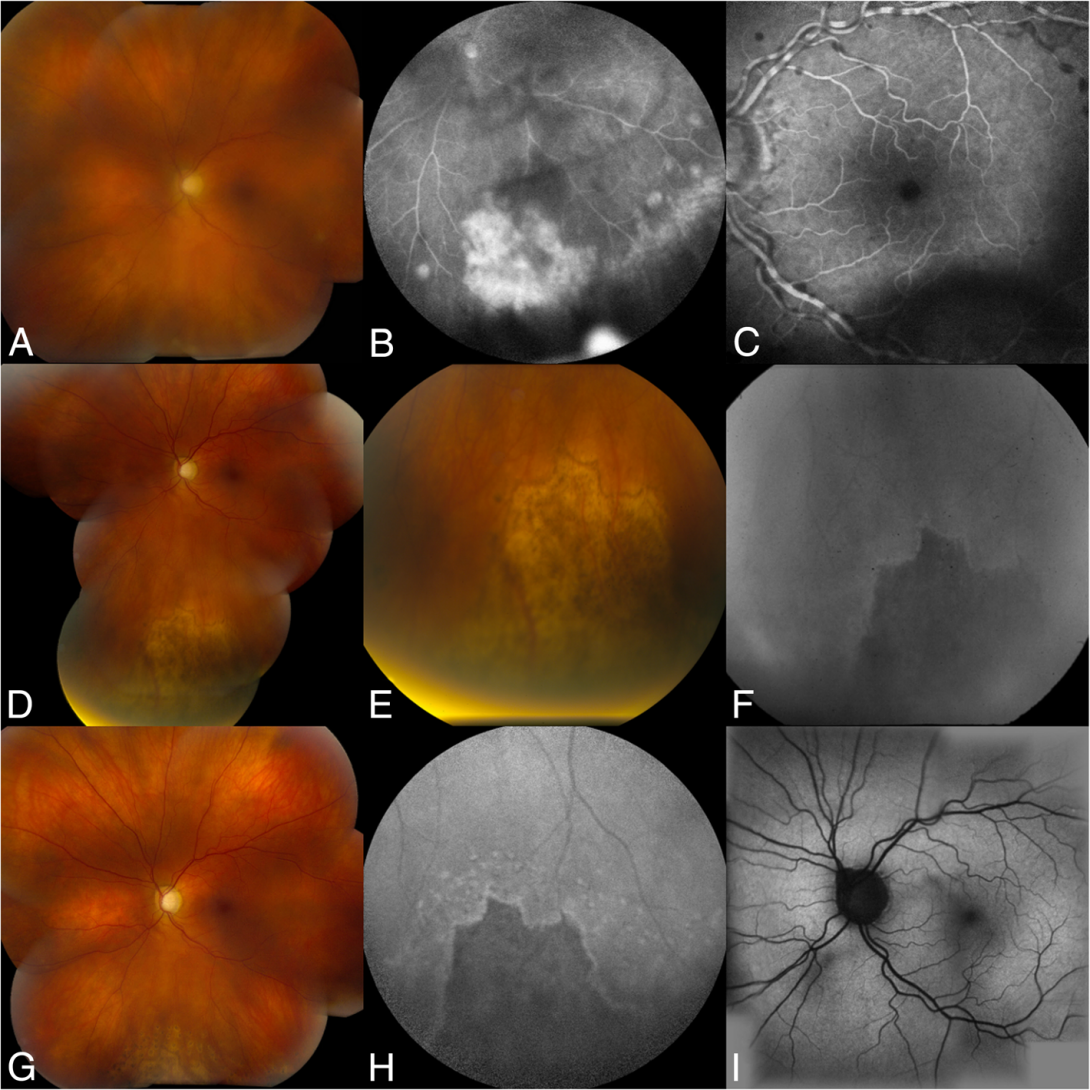
FAF imaging results. **(A-C)** Color fundus photograph and fluorescein angiography demonstrating presentation of an ARN lesion and vasculitis. **(D-F)** Color fundus photographs and FAF imaging demonstrating hyperautofluorescent borders adjacent to areas of complete retinal necrosis characterized by hypoautofluorescence 3 months following presentation. **(G-I)** Five months after presentation and after photocoagulation treatment color photos and fundus autofluorescence images demonstrate arrest of the disease margin in high contrast. The lesion margins were stable (unchanged) at 10 months.

Pinhole visual acuity was 20/25 + 1 right eye (OD) and 20/60 left eye (OS). There were no vesicular lesions or preauricular lymphadenopathy on examination. Slit lamp examination of the OD was unremarkable while the OS showed circumcorneal injection, mild anterior uveitis with moderate vitritis and haze. Fundus examination of the right eye was insignificant. Examination of the left eye was remarkable for vasculitis, a large area of wedge necrosis inferonasally from 6 to 7 o’clock and five small areas of necrosis temporally from approximately 3 to 5 o’clock with poorly defined margins (Figure 1).

The patient was clinically diagnosed with ARN and underwent anterior chamber paracentesis for viral polymerase chain reaction analysis, but there was insufficient fluid for analysis. She was admitted and started on IV acyclovir 600 mg Q8 h for 7 days [3]. Laboratory bloodwork revealed elevated VZV IgG, HSV1 IgG and cytomegalovirus (CMV) IgG levels. On day 3, the patient agreed to 0.1 ml Foscarnet (2.4 mg/0.1 mL) intravitreal injection OS [10,11]. On day 4, the retinitis appeared improved and she was started on oral prednisone 60 mg PO daily [3,12]. After completing a 7-day course of intravenous acyclovir, she was switched to valacyclovir 1,000 mg PO TID and she steadily improved.

Over the course of the disease, the patient was followed with serial FAF images. At 1 month following presentation, ocular inflammation was markedly improved and the vision stabilized at 20/25 OS. No inflammatory activity was ever noted in the OD. The patient had laser barricade performed 3 months following her presentation [3,13]. She was slowly weaned off of oral steroids 4 months following presentation. She continues to be followed on a maintenance dose of valacyclovir 1,000 mg PO QD without evidence of disease reactivation.

### Results and discussion

FAF changes have been reported in a case of PCR-proven varicella zoster-associated progressive outer retinal necrosis (PORN) and herpes simplex virus retinitis [13,14]. In this patient with ARN, we report imaging characteristics using FAF.

Hyperautofluorescent borders surrounding areas of hypoautofluorescence corresponding to outer retinal damage adjacent to complete retinal necrosis and RPE atrophy can be appreciated [8] (Figure 1). The high contrast seen at the FAF borders of retinitis allowed for more precise determination of the extent and progression of the disease than the color photos. Furthermore, it is important to note that the hyperautofluorescent border by itself does not indicate active inflammation; it merely permits better contrast on FAF. If on repeated FAF imaging the hyperautofluoroscent borders are seen to be extending posteriorly, then it may imply spreading or active inflammation. Our patient had active inflammation at presentation but the disease was arrested with management, thus limiting the extension of hyperautofluoroscent borders on follow-up FAF. The areas of retinal atrophy seen on color fundus photography that previously demonstrated active retinitis on FA showed persistent hypoautofluorescence. The hyperautofluoroscent pattern seen at the borders in early photos does follow the same pattern as reported earlier in a case report of PORN [14]. However, these results do not corroborate previous findings published regarding FAF and posterior uveitis in which Reznicek et al*.* reported a larger area of involvement on FAF than color fundus images [15]. This could be explained because of photos being captured at different time frames or persistent active inflammation in the two HSV retinitis cases reported [15]. Larger case series of FAF imaging in ARN will provide further insights to its usefulness in management of the disease but is difficult given the rarity of the disease.

### Conclusion

FAF permits visualization of a higher contrast border than color photos to help delineate lesions in ARN more accurately. The areas of retinal atrophy seen on color fundus photography that previously demonstrated active retinitis on FA showed persistent hypoautofluorescence. The borders of disease activity in ARN correlate with a high-contrast change in autofluorescence patterns that can be used to facilitate monitoring of disease progression.

### Consent

The patient has consented for the report to be published.

## Abbreviations

ARN: acute retinal necrosis
CMV: cytomegalovirus
FAF: fundus autofluorescence
HSV: herpes simplex virus
HIV: human immunodeficiency virus
PCR: polymerase chain reaction
PCR: retinal pigment epithelium
VZV: varicella zoster virus
